# Supplementary material for: Which Gelatin and Antibiotic Should Be Chosen to Seal a Woven Vascular Graft?
Source: Int J Mol Sci. 2024 Jan 12;25(2):965. doi: 10.3390/ijms25020965 (PMC10816219; doi:10.3390/ijms25020965)
Supplement: Supplementary file 1 [file ijms-25-00965-s001.zip › ijms-2717783-supplementary.pdf]

Supplementary Data

Which gelatin and antibiotic should be chosen to seal a woven vascular graft?

Irina Y. Zhuravleva*, Aldar A. Shadanov, Maria A. Surovtseva, Andrey A. Vaver, Larisa M. Samoylova, Sergey V.Vladimirov, Tatiana P. Timchenko, Irina I.Kim, Olga V. Poveschenko

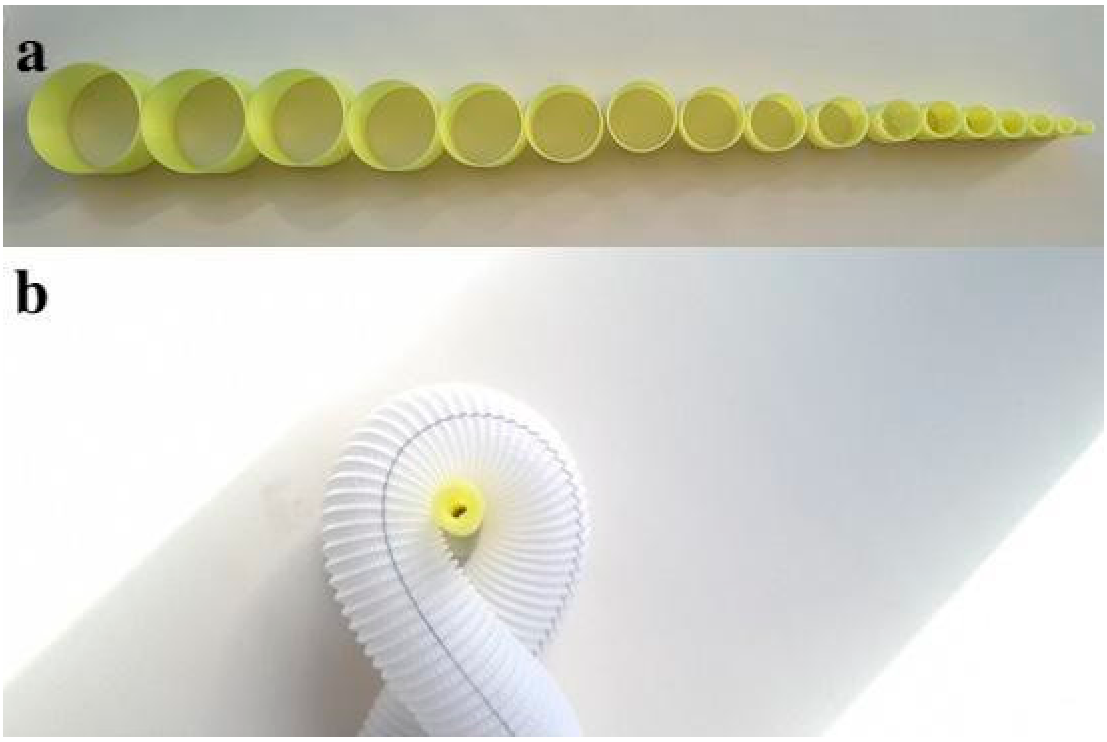

Figure S1. Kinking radius measurement according to ISO 7198:2016. a – cylindrical calibers with a radius from 4 to 42.5 mm in increments of 1.5 mm. b - making a prosthesis loop, cylindrical caliber is 8 mm in diameter (r = 4 mm).

Table S1

The statistical significance of the differences in EAhy 926 cell viability under the influence of studied samples' extracts after 72 hours

	1	2	3	4	5	6	7	8
2	0.144							
3	0.012	0.095						
4	0.012	0.037	0.403					
5	0.012	0.012	0.012	0.012				
6	0.011	0.011	0.011	0.011	0.011			
7	0.012	0.012	0.011	0.012	0.753	0.011		
8	0.012	0.012	0.012	0.012	0.095	0.139	0.296	
0	0.020	0.020	0.027	0.178	0.270	0.018	0.111	0.027

Table S2

Statistical significance of differences in the number of adhered EAhy926 cells after 3 days of culture

	1	2	3	4	5	6	7	8
2	0.020							
3	0.011	0.011						
4	0.012	0.238	0.012					
5	0.688	0.011	0.011	0.011				
6	0.176	0.012	0.011	0.024	0.270			
7	0.030	0.011	0.011	0.012	0.283	0.085		
8	0.030	0.027	0.012	0.011	0.403	0.076	0.176	
0	0.020	0.030	0.025	0.021	0.375	0.830	0.168	0.540

Table S3

Statistical significance of differences in the number of adhered EAhy926 cells after 7 days of culture

	1	2	3	4	5	6	7	8
2	0.000							
3	0.001	0.290						
4	0.000	0.030	0.471					
5	0.020	0.004	0.004	0.004				
6	0.027	0.020	0.020	0.030	0.283			
7	0.020	0.030	0.300	0.030	0.117	0.340		
8	0.004	0.001	0.030	0.005	0.027	0.283	0.283	
0	0.004	0.001	0.001	0.001	0.004	0.004	0.001	0.010

Table S4

Statistical significance of differences in the number of adhered EAhy926 cells after 14 days of culture

	1	2	3	4	5	6	7	8
2	0.037							
3	0.030	0.340						
4	0.001	0.797	0.416					
5	0.014	0.340	0.013	0.013				
6	0.117	0.340	0.020	0.020	0.416			
7	0.000	0.097	0.060	0.060	0.000	0.020		
8	0.000	0.340	0.060	0.003	0.085	0.060	0.013	
0	0.143	0.013	0.004	0.004	0.001	0.085	0.010	0.010

Table S5

The statistical significance of the differences in cell viability after 3 days cultivation

	1	2	3	4	5	6	7	8
2	0.030							
3	0.059	0.059						
4	0.030	0.309	0.309					
5	0.029	0.110	0.030	0.030				
6	0.029	0.110	0.061	0.309	0.309			
7	0.030	0.059	0.061	0.030	0.081	0.081		
8	0.030	0.061	0.030	0.030	0.059	0.061	0.081	
0	0.030	0.030	0.029	0.030	0.059	0.029	0.059	0.081

Table S6

The statistical significance of the differences in cell viability after 7 days cultivation

	1	2	3	4	5	6	7	8
2	0.029							
3	0.029	0.081						
4	0.029	0.030	0.312					
5	0.029	0.030	0.061	0.030				
6	0.059	0.061	0.061	0.112	0.312			
7	0.312	0.770	0.312	0.029	0.061	0.061		
8	0.028	0.312	0.059	0.030	0.061	0.770	0.309	
0	0.029	0.030	0.030	0.030	0.030	0.030	0.030	0.029

Table S7

The statistical significance of the differences in cell viability after 14 days cultivation

	1	2	3	4	5	6	7	8
2	0.194							
3	0.061	0.061						
4	0.029	0.029	0.030					
5	0.030	0.030	0.030	0.312				
6	0.059	0.061	0.312	0.312	0.061			
7	0.030	0.030	0.061	0.029	0.030	0.312		
8	0.061	0.061	0.059	0.030	0.030	0.312	0.194	
0	0.030	0.030	0.030	0.029	0.030	0.029	0.030	0.030

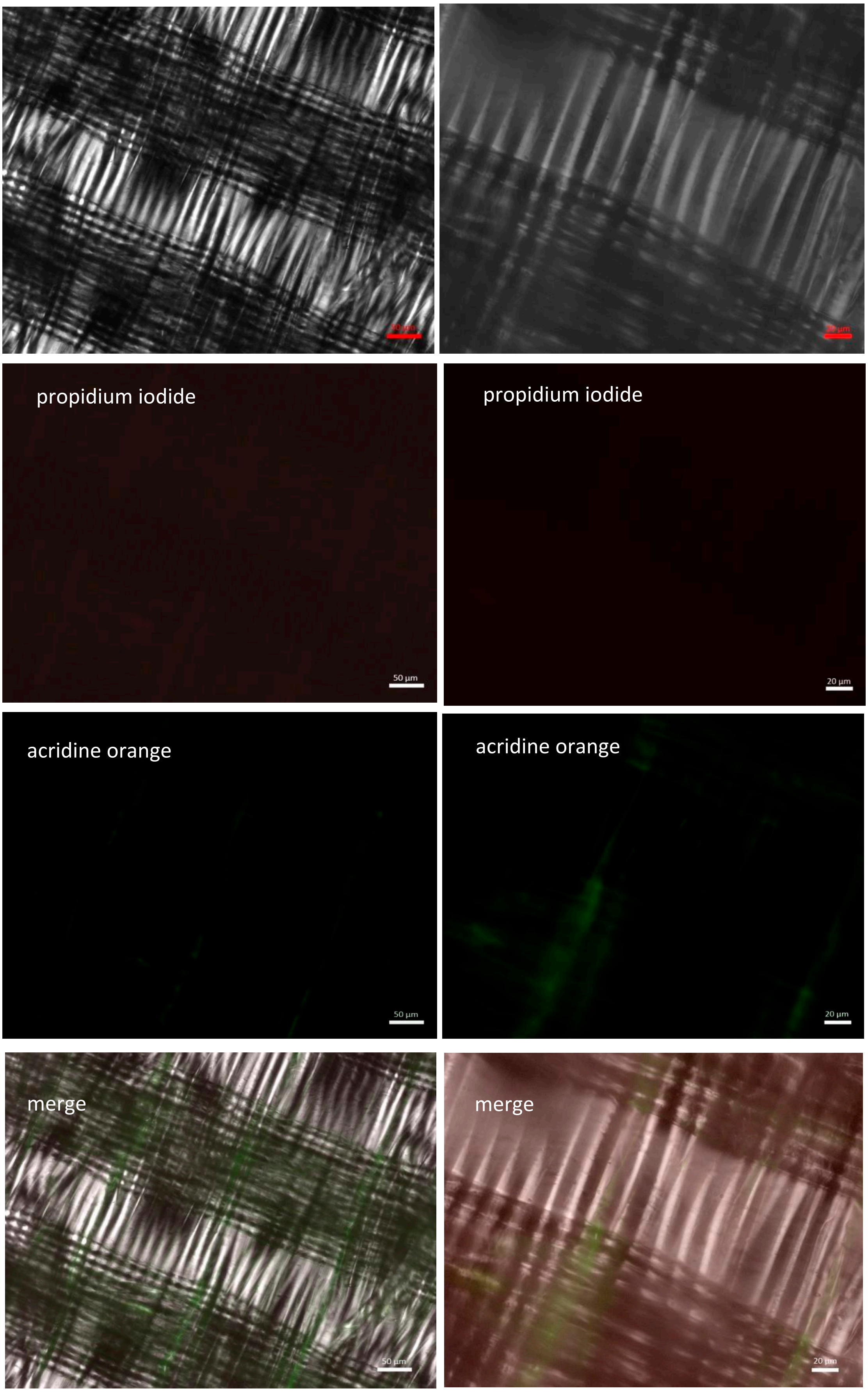

Figure S2. *Fluorescence microscopy images of a cell-free vascular woven graft sealed with gelatin A + vancomycin (No. 3). Staining was performed with acridine orange (green) and propidium iodide (red). The scale bars are 50 µm for the left column and 20 µm for the right column.*

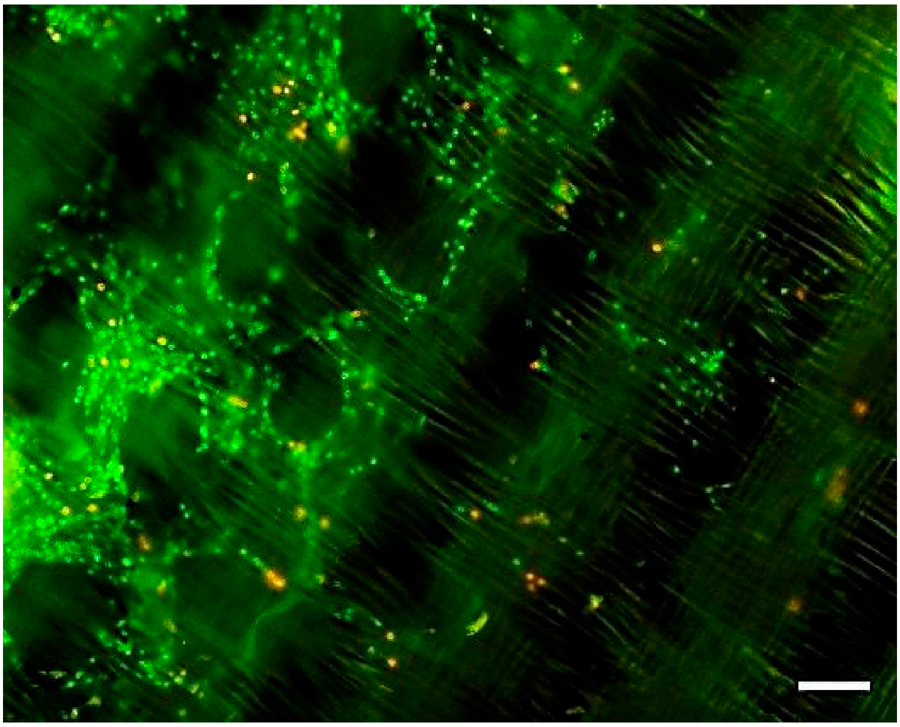

Figure S3. Fluorescence microscopy images of tube formation from endothelial cells on sample No.6 (gelatin B + Vancomycin) after seven days of culture. Staining with acridine orange (green, live cells) and propidium iodide (red, dead cells). The scale bar is 100 µm

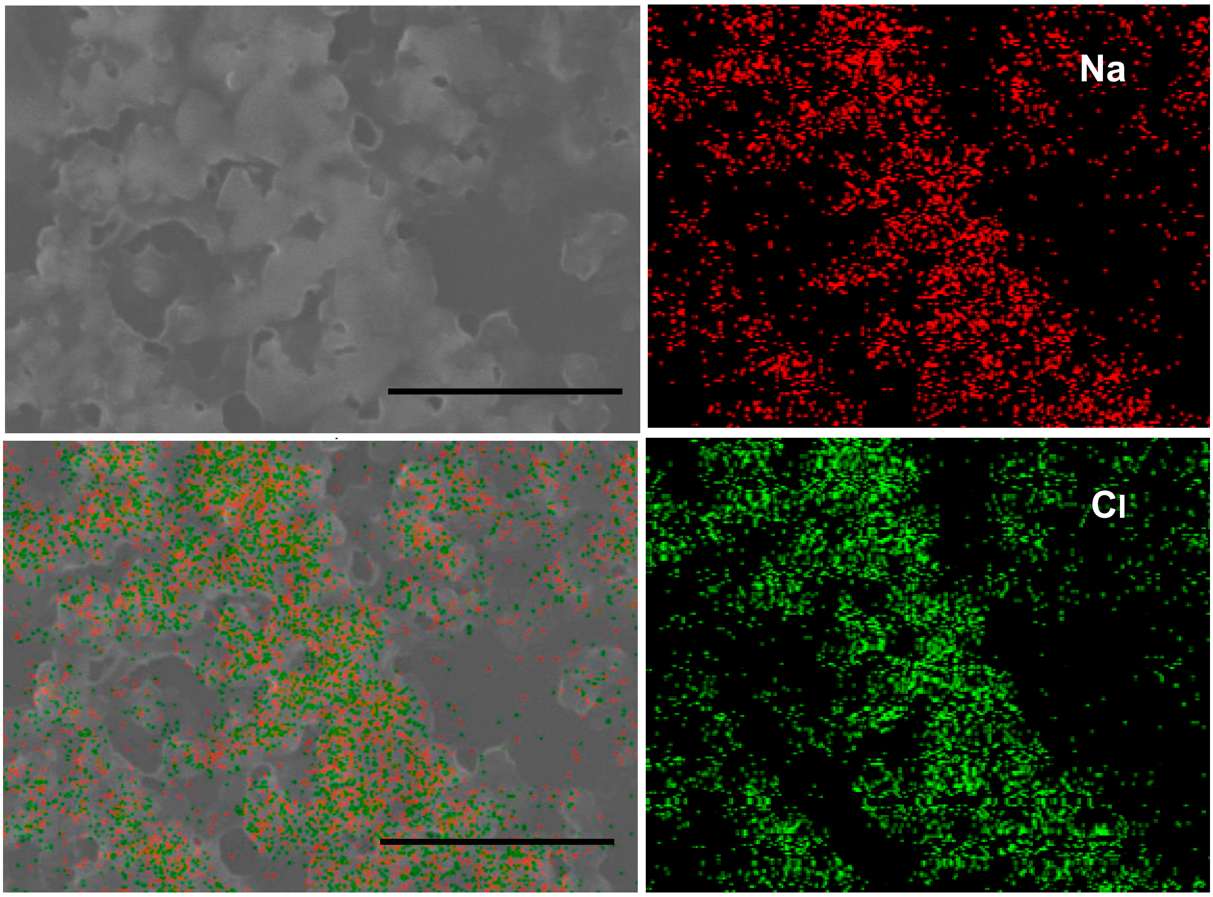

Figure S4. Left column: SEM image of the mouse fibroblast cell culture on glass (top) and merged image (bottom). Left column: distribution of sodium (Na) and chlorine (Cl). The scale bar is 10 µm.

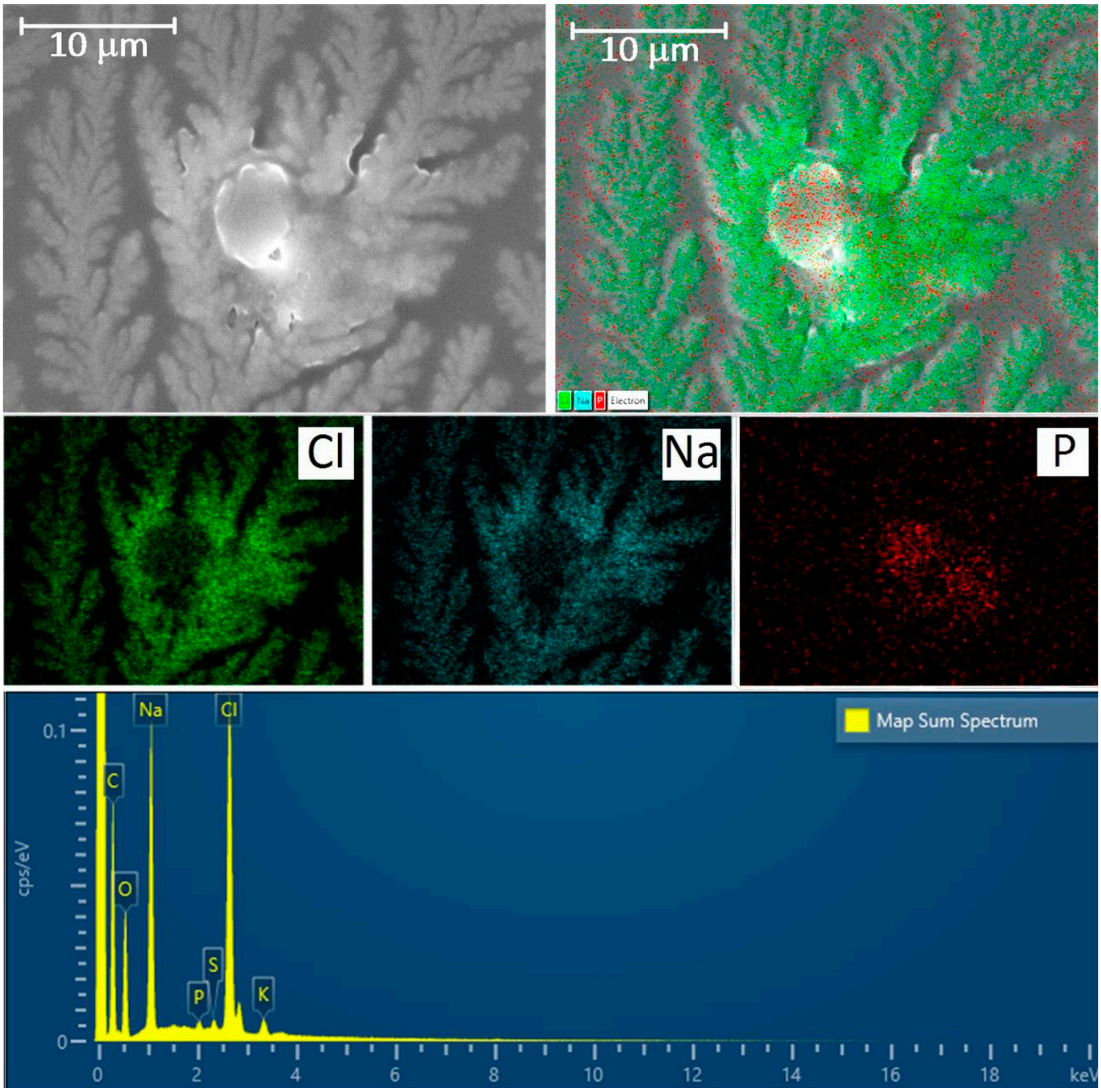

Figure S5. Top row: SEM image of the mouse fibroblasts in a NaCl-containing culture medium and merged image (B). Middle row: distribution of chlorine (Cl), sodium (Na), and phosphorus (P). Na and Cl distribution corresponds to salt crystals formed during the culture medium drying. The distribution of phosphorus corresponds to the cells' localization. Bottom row: EDS spectrum of the observation field. The scale bar is 10 μm.

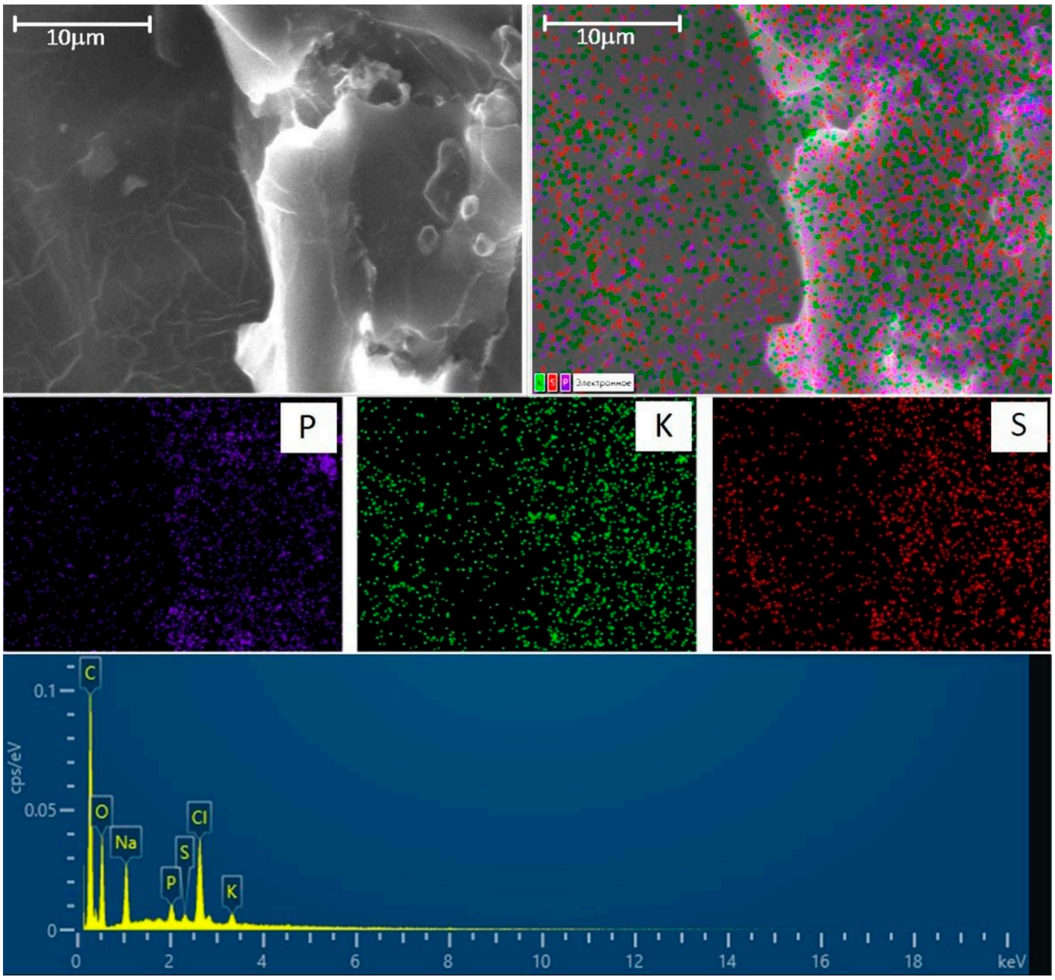

Figure S6. Top row: SEM image of the graft wall cross-section and merged image (B). Middle row: distribution of phosphorus (P), potassium (K), and sulfur (S). Bottom row: EDS spectrum of the observation field. Scale bar 10 μm.
